# Nicotinamide in the prevention of breast cancer recurrences?

**DOI:** 10.18632/oncotarget.27173

**Published:** 2019-09-17

**Authors:** Giulia Dell’Omo, Paolo Ciana

**Affiliations:** Department of Oncology and Hemato-Oncology, University of Milan, Milan 20133, Italy

**Keywords:** chemoprevention, SIRT1 inhibitors, mammary ­cancer, NSAIDs

The estimate of new cancer cases reached 18.1 million worldwide in 2018 [1], with a constantly growing trend that needs to be contrasted by efficacious (chemo)preventive strategies able to counteract the dreadful global burden of this disease [2]. Several preclinical, epidemiological and clinical studies proposed non-steroidal anti-inflammatory drugs (NSAIDs) as one of the most promising class of compounds for the prevention of different cancer types, including mammary tumors [3], even if their use in long-term treatment is limited by side effects due to cyclooxygenase (COX) inhibition.

Recently, we identified SIRT1 as a novel target for NSAID chemopreventive activity, independent from COX [4]. SIRT1 is the mammalian homolog of yeast silent information regulator 2 (Sir2), member of the NAD^+^-dependent class III histone deacetylases. SIRT1 deacetylates various protein substrates including histones, DNA-repair, intracellular signaling and transcription factors; because of this pleiotropic action, SIRT1 has been implicated in the regulation of several biological processes related with cancer such as energetic metabolism, proliferation, inflammation, apoptosis and hypoxia [5]. Our work demonstrated that NSAIDs with different chemical structures can directly interact with the enzyme and inhibit its deacetylase activity. Because of the SIRT1 inhibition, the oncosuppressor p53/P21 pathway is activated and counteracts the cell hyperproliferation occurring during initial tumorigenesis steps in preclinical breast cancer models and in patients [4]. Although several studies reported a controversial role for SIRT1 inhibition in tumorigenesis, a recent phase III clinical trial investigating the effects of nicotinamide (vitamin B3, the physiological inhibitor of SIRT1) in non-melanoma skin cancer demonstrated the ability of this vitamin to reduce the rates of new tumorigenic events in high-risk patients characterized by immunosuppression and DNA repair defect. In these patients, nicotinamide administration increased the level of pro-inflammatory cytokines and stimulated DNA repair, thus significantly reducing the risk of tumor onset [6]. This clinical result, substantiated by several preclinical studies [7], points to SIRT1 inhibition as a potential strategy for cancer prevention using a candidate drug of natural origin, nicotinamide, particularly attractive for a non-toxic, cheap chemopreventive treatment.

Our finding demonstrating the ability of NSAIDs to inhibit SIRT1 and activate p53/P21 signaling is particularly intriguing in light of recent epidemiological data associating the perioperative administration of ketorolac and diclofenac with a significant reduction of relapse events in breast and ovary cancer patients [8, 9], especially for those with increased BMI [10]. The repurposing of these NSAIDs in cancer prevention is currently under consideration in clinical trials designed to support the epidemiological findings. In our work, we observed that in breast cancer patients receiving ketorolac as antalgic treatment during mastectomy, the SIRT1-p53 pathway is selectively activated by the NSAID administration [4], suggesting a direct association between SIRT1 inhibition and the reduction of recurrence risk in these patients.

**Figure 1:**
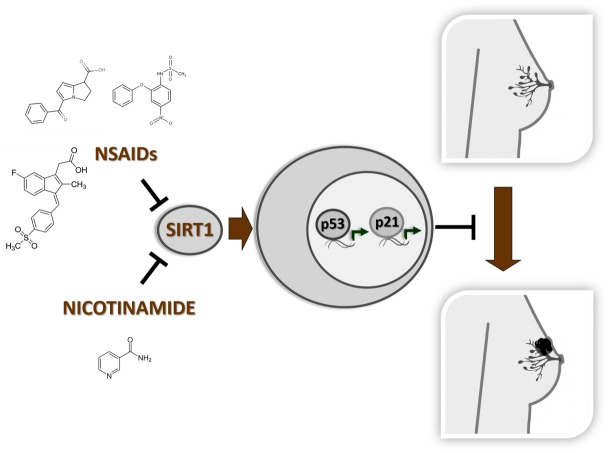
Nicotinamide and NSAIDs are binding and inhibiting SIRT1 deacetylase. The enzymatic inhibition by these molecules stimulates the anti-proliferative p53/P21 pathway reducing the risk of invasive breast cancer insurgence.

The clinical relevance of the p53/P21 pathway stimulation produced by SIRT1 inhibitors in the active prevention of mammary cancer has never been investigated. Our study provides the rational basis for a prospective clinical trial aiming at the evaluation of nicotinamide effects on p53/P21 pathway and relapse risk in breast cancer patients undergoing mastectomy (Figure 1). Using nicotinamide as SIRT1 inhibitor, it would be possible to evaluate the effects of the long-term enzymatic inhibition after mastectomy that is expected to reduce the relapse risk to a major extent compared to the perioperatory ketorolac or diclofenac administration. In this sense, nicotinamide represents a better candidate drug respect to NSAIDs, whose sub-optimal safety profile limits their use only to short-term treatments (e.g. during the antalgic therapy in the surgery procedure). In conclusion, our study casts the evidence for the chronic use of vitamin B3 after mastectomy to actively prevent the progression into a life-threatening disease; moreover, this can be an initial proof-of-principle for a wider application of nicotinamide treatment in cancer chemoprevention beyond breast and skin cancer fields.
